# Effect of interleukin-1β treatment on co-cultures of human meniscus cells and bone marrow mesenchymal stromal cells

**DOI:** 10.1186/1471-2474-14-216

**Published:** 2013-07-22

**Authors:** Anika Chowdhury, Louis W Bezuidenhout, Aillette Mulet-Sierra, Nadr M Jomha, Adetola B Adesida

**Affiliations:** 1 Department of Surgery, Division of Orthopaedic Surgery, Laboratory of Stem Cell Biology and Orthopaedic Tissue Engineering, University of Alberta, Edmonton, AB T6G 2E1, Canada

**Keywords:** Bone marrow stromal cells, Chondrogenesis, Co-cultures, Fibrochondrogenesis, Meniscus cells, Meniscus, Tissue engineering

## Abstract

**Background:**

Interleukin-1β (IL-1β) is a major mediator of local inflammation present in injured joints. In this study, we aimed at comparing the effect of IL-1β on engineered tissues from MCs, BMSCs and co-cultured MCs and BMSCs.

**Methods:**

We compared the effect of IL-1β in 3 groups: (1) MCs, (2) BMSCs and, (3) co-cultures of MCs and BMSCs. We selected 1 to 3 ratio of MCs to BMSCs for the co-cultures. Passage two (P2) human BMSCs were obtained from two donors. Human MCs were isolated from menisci of 4 donors. Mono-cultures of MCs and BMSCs, and co-cultures of MCs and BMSCs were cultured in chondrogenic medium with TGFβ3, as cell pellets for 14 days. Thereafter, pellets were cultured for 3 more days in same medium as before with or without IL-1β (500 pg/ml). Pellets were assessed histologically, biochemically and by RT-PCR for gene expression of aggrecan, sox9, MMP-1, collagens I and II. Statistics was performed using one-way ANOVA with Tukey’s post-tests.

**Results:**

Co-cultured pellets were the most intensely stained with safranin O and collagen II. Co-cultured pellets had the highest expression of sox9, collagen I and II. IL-1β treatment slightly reduced the GAG/DNA of co-cultured pellets but still exceeded the sum of the GAG/DNA from the proportion of MCs and BMSCs in the co-cultured pellets. After IL-1β treatment, the expression of sox9, collagen I and II in co-cultured pellets was higher compared to their expression in pure pellets. IL-1β induced MMP-1 expression in mono-cultures of MCs but not significantly in mono-cultures of BMSCs or in co-cultured pellets. IL-1β induced MMP-13 expression in mono-cultured pellets of BMSCs and in co-cultured pellets.

**Conclusions:**

Co-cultures of MCs and BMSCs resulted in a synergistic production of cartilaginous matrix compared to mono-cultures of MCs and BMSCs. IL-1β did not abrogate the accumulated GAG matrix in co-cultures but mediated a decreased mRNA expression of aggrecan, collagen II and Sox9. These results strengthen the combinatorial use of primary MCs and BMSCs as a cell source for meniscus tissue engineering by demonstrating retention of fibrochondrogenic phenotype after exposure to IL-1β.

## Background

The meniscus is a semilunar-shaped fibrocartilaginous tissue located between the femur and the tibia [1,2]. It is essential for mechanical load distribution, joint stability and protection of articular cartilage in the knee joint [3-6]. The biomechanical role of the meniscus is a result of its extracellular matrix (ECM) [7]. The ECM is synthesized entirely by resident meniscus fibrochondrocytes and it is composed of type I collagen throughout the entire tissue, and type II collagen and proteoglycans in the avascular inner two-thirds region of the tissue [8,9]. Perhaps as a consequence of the tissue’s constrained blood supply, the reparative and regenerative capacity of the meniscus is limited and injuries to the avascular portion naturally do not heal. Depending on the extent of avascular meniscus injuries, partial or total meniscectomy is performed which is a common procedure. While these surgeries may alleviate meniscus injury related pain, they fail to restore normal joint biomechanics and are associated with high incidence of early development of degenerative joint disease, such as osteoarthritis (OA) [10-12]. Thus, there is much interest in the development of alternative, biological approaches to meniscus repair and regeneration [13]. Among these, there is considerable interest in cell-based tissue engineering and regenerative medicine strategies with the view to generate functional meniscus substitutes to replace or aid repair of damaged menisci [14-29]. However, the optimal cell source for these approaches is yet to be determined [20,24,30]. Naturally, meniscus cells are the obvious choice but the use of these cells have several drawbacks that include insufficient numbers of differentiated meniscus cells and loss of ECM-forming phenotype of *in vitro* multiplied meniscus cells [24]. Bone marrow mesenchymal stromal cells (BMSCs) have also been explored as a cell source for meniscus tissue engineering with the outcome of forming a meniscus-like fibrocartilage [30,31]. However, BMSCs are susceptible to undergoing hypertrophic differentiation [32]. Recent findings in our laboratory and others demonstrated that co-culture of primary human meniscus cells with BMSCs in the presence of chondrogenic factors resulted not only in a synergistically enhanced production of meniscus-like ECM and fibrocartilage tissue-like formation, but additionally the suppression of hypertrophic differentiation of BMSCs [21,33,34]. Although the mechanism underlying the synergistic matrix production is to be explored, the interplay of primary meniscus cells and BMSCs offers the perspective of delivering a combinatorial cell source for meniscus reconstruction, with the benefit of retention of the matrix-forming phenotype of differentiated meniscus cells and enhanced functional matrix production. However, for the combination of primary human meniscus cells and BMSCs to be considered as a cell source for the generation of functional meniscal grafts, it is important to evaluate their response to mediators of inflammation, which are typically present in injured joints or as a consequence of the iatrogenic trauma of the meniscus repair surgery itself. Pro-inflammatory cytokines, such as interleukin-1β (IL-1β), are major mediators of local inflammation and are known to be present in injured joints. In the present study, we aimed at studying the effect of IL-1β on engineered tissues from meniscus cells (MC), BMSCs and co-cultured MCs and BMSCs. We compared the effect of IL-1β in three study groups: (1) MCs, (2) BMSCs and, (3) co-cultures of MCs and BMSCs. For the co-cultured cell group, we selected a 1 to 3 ratio of MCs to BMSCs. Our previous work showed that this ratio reproducibly resulted in synergistic matrix formation after *in vitro* chondrogenic differentiation in 3D culture using the pellet model of mesenchymal cell condensation [21]. We hypothesized that co-cultured MCs and BMSCs will retain an enhanced chondrogenic matrix-forming capacity compared to mono-cultured MCs and mono-cultured BMSCs after short-term treatment with IL-1β.

## Methods

### Collection of bone marrow specimens and culture of bone marrow stem cells

Local ethical committee approval of the University of Alberta, Edmonton, Canada was obtained for this study. Bone marrow aspirates were acquired during routine orthopaedic procedures from the iliac crest of two male donors (age 45 and 57 years). The number of mononucleated cells (MNCs) in the aspirates was determined by crystal violet nuclei staining and cell counting on a haemocytometer. Thereafter, 15 million MNCs were seeded per 150 cm^2^ tissue culture flask. Culture media was alpha MEM supplemented with 10% heat inactivated fetal bovine serum, 1 mM sodium pyruvate, 100 mM HEPES buffer, 1 mM sodium pyruvate, 100 U/ml penicillin, 100 μg/ml streptomycin, 0.29 mg/ml L-glutamine (all from Invitrogen, Mississauga, Ontario, Canada) and 5 ng/ml of basic FGF or FGF-2 (Neuromics, Edina, MN, USA). Plastic adherent MNCs were allowed to attach and proliferate for 7 days before the first media change under normal oxygen tension (21% O_2_; 95% air) at 37°C in a humidified incubator with 5% CO_2_. Thereafter, media change was implemented twice per week until 70-80% cell confluence was attained. The plastic adherent MNCs populations now termed bone marrow mesenchymal stromal cells (BMSCs) were detached using trypsin-EDTA (0.05% w/v) and expanded until passage 2 prior to experimental use. We characterized the BMSCs as we have previously described using a panel of cell surface markers and flow cytometry analysis [35].

### Human menisci and meniscus cells isolation

Local ethical committee approval of the University of Alberta, Edmonton, Canada was obtained to acquire menisci for this study. Both lateral and medial menisci were harvested from the knee joint of 4 male donors (age 56–76, mean age 66 ± 9 years) undergoing total knee arthroplasty because of osteoarthritis. Meniscus cells (MCs) were released via treatment with type II collagenase (0.15% w/v; Worthington, Lakewood, NJ, USA) after 16 h digestion of tissue at 37°C in a standard medium- high glucose Dulbecco’s modified Eagle’s medium containing 4.5 mg/ml D-Glucose (DMEM-HG), 0.1 mM non-essential amino acids, 1 mM sodium pyruvate, 100 mM HEPES buffer, 1 mM sodium pyruvate, 100U/ml penicillin, 100 μg/ml streptomycin, 0.29 mg/ml L-glutamine supplemented with 10% FBS - (all from Invitrogen, Mississauga, Ontario, Canada). The cell suspension obtained after digestion was passed through a 70 μm nylon-mesh filter. Isolated cells were plated at 10^4^ cells/cm^2^ and cultured in standard medium for 48 h under normal oxygen tension (21% O_2_; 95% air) at 37°C in a humidified incubator with 5% CO_2_ before experimental use. Non-adherent cells were aspirated off while adherent cells were detached with trypsin-EDTA (0.05% w/v) (from Invitrogen).

### Chondrogenic differentiation in pellet cultures

Mono-cultures of MCs and BMSCs in the form of pellets were formed in 250 μl of serum-free chondrogenic culture medium consisting of standard medium supplemented with 0.1 mM ascorbic acid 2-phosphate, 10^-5^ M dexamethasone, 1× ITS + 1 premix (Sigma-Aldrich, Oakville, Canada), 10 ng/ml TGF-β3 (Humanzyme-Medicorp Inc.) as described previously [36]. A total of 2.5 × 10^5^ cells were spun in 1.5 ml sterile conical polypropylene microfuge tubes (Enzymax LLC, Kentucky, USA) at 1500 rpm (433 g) for 3 minutes to form spherical cell pellets. Co-cultures in pellet form consisting of MCs: BMSCs were formulated by mixing the two cell types at a 1:3 ratio, respectively. Briefly, for each co-cultured pellet we mixed 62, 500 primary (unexpanded) meniscus cells with 187, 500 BMSCs at passage 2 (*in vitro* expanded cells). Primary meniscus cells from two donors were co-cultured with BMSCs at passage 2 from one donor and primary meniscus cells from the other two others were co-cultured with BMSCs at passage 2 from the other bone marrow aspirate donor. Control pellets were formed from either pure primary meniscus cells or pure BMSC (at passage 2) at cell density of 250,000 per pellet. Previous studies had shown that the co-cultured cell-cell ratio reproducibly resulted in enhanced matrix formation [21]. For each group, a minimum of six pellets were formed for subsequent histological, biochemical and molecular analysis. After 2 weeks of pellet culture with media changes 2 times per week, the pellets were cultured for 3 additional days in chondrogenic culture medium in the presence (i.e. MC+, BMSC + and MC:BMSC+) or absence (i.e. MC, BMSC and MC:BMSC) of 500 pg/ml of interleukin-1β (Humanzyme-Medicorp Inc.). After the culture period, pellets were processed biochemically for GAG and DNA contents, histologically, immuno-histochemically and by real-time quantitative RT-PCR for gene expression analyses. For each experimental repeat (4 in total), there was 1 experimental group (co-culture of MC:BMSC+) and 5 control groups (1 co-culture of MC:BMSC and 4 mono-cultures of BMSC; BMSC+; MC; MC+). In total there were 24 pellets per each group.

### Biochemical analysis

Pellets were rinsed in phosphate buffer saline (PBS; Invitrogen) prior to 16 h digestion in 250 μl of proteinase K (1 mg/ml in 50 mM Tris with 1 mM EDTA, 1 mM iodoacetamide and 10 mg/ml pepstatin A – all from Sigma-Aldrich) at 56°C. The media collected in the last 3 days of pellet culture was analysed for GAG content. GAG contents in pellets and the collected media were measured spectrophotometrically after reaction with 1,9-dimethylmethylene blue binding (Sigma-Aldrich), with chondroitin sulfate (Sigma-Aldrich) as a standard [37]. The DNA content was determined spectrofluorometrically using the CyQuant cell proliferation assay kit (Invitrogen) with supplied bacteriophage λ DNA as standard. Based on experimental GAG per DNA values of mono-cultures of BMSC or MC pellets, the calculated GAG per DNA values were calculated as a linear function of the proportion (%) of BMSCs and MCs using the following equations [36]:

$$\begin{array}{l} {\mathrm{GAG}/\mathrm{DNA}}_{\text{calculated}}=[{\left(\mathrm{GAG}/\mathrm{DNA}\right)}_{100\%\mathrm{BMSC}}\\ \;\times 75\%\mathrm{BMSC}+{\left(\mathrm{GAG}/\mathrm{DNA}\right)}_{100\%\mathrm{MC}}\\ \;\times 25\%\mathrm{MC}] \end{array}$$

If the calculated GAG/DNA is significantly less than the experimentally determined GAG/DNA value of co-cultured pellets, then a synergistically enhanced chondrogenic GAG matrix formation is considered to have taken place [36].

### Histology and Immuno-histochemical analyses

Tissues generated from the pellet cultures were fixed in 4% (v/v) phosphate buffered formalin, processed into paraffin wax, sectioned at 5 μm and stained with 0.1% (w/v) Safranin O and counterstained with 1% (w/v) Fast Green stain, to reveal sulfated proteoglycan (GAG) matrix depositions. Other sections were probed with antibodies raised against collagen types I and II. Sections were digested with trypsin and then incubated with antibodies against collagen II (II-II6B3) from Developmental Studies Hybridoma Bank at University of Iowa, USA. Immuno-localised antigens were visualized with goat anti-mouse IgG biotinylated secondary antibody (Dako Canada Inc., Mississauga, Ontario, Canada) and a streptavidin-horseradish peroxidase labeling kit with 3,3′-diaminobenzidine (Dako). Images were captured on an Omano OM159T biological trinocular microscope (Microscope Store, Virginia, USA) fitted with an Optixcam summit series 5MP digital camera and Optixcam software and assembled in Adobe Photoshop (Adobe Systems Inc. San Jose, USA).

### Real-time quantitative RT-PCR assays

Total RNA was extracted from pellets using Trizol (Invitrogen) after grinding with Molecular Grinding Resin (Geno Technology Inc. St Louis, USA) in combination with the use of RNeasy mini kit (Qiagen, Mississauga, Ontario, Canada) and after removal of contaminating genomic DNA from the pellets by DNase treatment. To minimize changes in gene expression during cell pellet harvest, cell pellets were immediately (< 1 min) transferred into Trizol. Total RNA (100 ng) in a 40 μl reaction was reverse transcribed to cDNA using GoScript reverse transcriptase) primed with oligo(dT)_15_ primer (Fisher Scientific, Whitby, Ontario, Canada). Quantitative real-time polymerase chain reaction (qRT-PCR) was performed in DNA Engine Opticon II Continuous Fluorescence Detection System (Bio-Rad) using hot start Taq and SYBR Green detection (Eurogentec North America Inc., San Diego, CA, USA). Primer sequences for aggrecan (AGG), collagen I (*COL1A2*), collagen II (*COL2A1*), collagen X (*COL10A1*), interleukin 1 receptor antagonist (IL1Ra), matrix metalloproteinase −1 (MMP-1), matrix metalloproteinase −2 (*MMP-2*), matrix metalloproteinase −13 (*MMP-13*), SOX9 and β-actin (Table 1) were taken from previously published work or were custom designed using the Primer Express software (Applied Biosystems, Foster City, USA) [38-40]. All primers were obtained from Invitrogen. For each cDNA sample, the threshold cycle (Ct) value of each target gene was reduced by subtraction of Ct value of human β-actin to derive ΔCt. The level of gene expression was calculated as 2-^Δct^[41]. Each sample was assessed at least in triplicates for each gene of interest.

**Table 1:** Primer sequences used in quantitative real-time PCR (all primers were purchased from Invitrogen, Mississauga, Ontario, Canada)

**Gene**	**Primer**	**Direction**
β-Actin	5′-AAGCCACCCCACTTCTCTCTAA-3′	(Forward)
	5′-AATGCTATCACCTCCCCTGTGT-3′	(Reverse)
Aggrecan	5′-AGGGCGAGTGGAATGATGTT-3′	(Forward)
	5′-GGTGGCTGTGCCCTTTTTAC-3′	(Reverse)
Collagen I (*COL1A2*)	5′-TTGCCCAAAGTTGTCCTCTTCT-3′	(Forward)
	5′-AGCTTCTGTGGAACCATGGAA-3′	(Reverse)
Collagen II (*COL2A1*)	5′-CTGCAAAATAAAATCTCGGTGTTCT-3′	(Forward)
	5′-GGGCATTTGACTCACACCAGT-3′	(Reverse)
Collagen 10A1 (*COL10A1*)	5′-GCCTCACTTATTAAAGCACAAAATGT-3′	(Forward)
	5′-AATGGTTGAGAACAGCAAATTGC-3′	(Reverse)
IL1Ra	5′-CTGCACAGCGATGGAAGCT-3′	(Forward)
	5′-GCCTTCGTCAGGCATATTGG-3′	(Reverse)
MMP-1	5′-ATGAGTCTTTGCCGGAGGAA-3′	(Forward)
	5′-GTGACACCAGTGACTGCACATG-3′	(Reverse)
MMP-3	5′-CATCCAAAAACGCCAGACAA-3′	(Forward)
	5′-CGGAGACTGGTAATGGCATCA-3′	(Reverse)
MMP-13	5′-CATCCAAAAACGCCAGACAA-3′	(Forward)
	5′-CGGAGACTGGTAATGGCATCA-3′	(Reverse)
*SOX9*	5′-CTTTGGTTTGTGTTCGTGTTTTG-3′	(Forward)
	5′-AGAGAAAGAAAAAGGGAAAGGTAAGTTT-3′	(Reverse)

### Statistical analysis

For each experimental repeat and donor, at least triplicate specimens were assessed and the data were presented as mean ± standard error of mean (SEM) of measurements. All statistical analyses were performed using SPSS version 20 (IBM SPSS Statistics 20; Chicago, IL, USA) unless stated otherwise. Differences between experimental groups were assessed by one-way ANOVA with Tukey’s multiple comparison post-tests and considered significant with *p* < 0.05 (i.e. *p < 0.05, ** *p* < 0.01 and ***p < 0.001).

## Results

### Enhanced cartilaginous tissue formation in co-cultures of meniscus cells (MCs) and BMSCs

**Figure 1:**
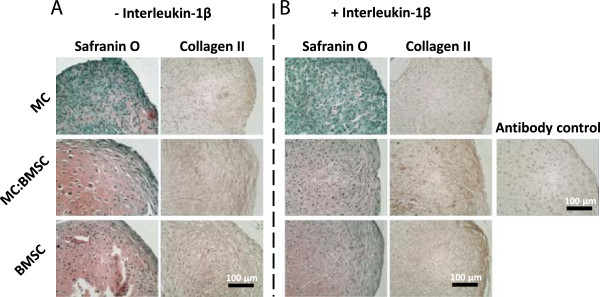
**Histological characteristics of pellets formulated from mono-cultured MCs, mono-cultured BMSCs and co-cultures of MC and BMSCs after a total of 17 days culture in defined serum-free chondrogenic media. (A-B)** Safranin O and collagen II immuno-histochemical staining of representative pellets from cells derived from the same donor. Magnification lens × 20; scale bar is 100 μm.

**Figure 2:**
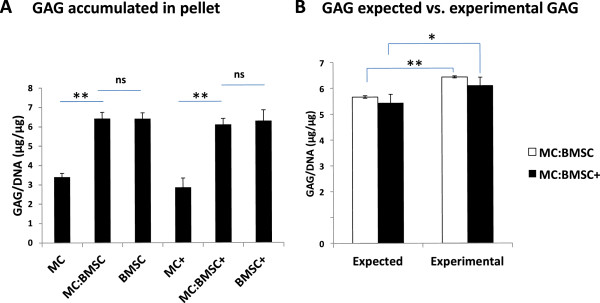
**Accumulation of cartilaginous matrix in cell pellets after a total of 17 days culture in serum-free chondrogenic media. (A)** Accumulated glycosaminoglycan (GAG) matrix content of cell pellets normalized to DNA amount. Data are mean ± standard error of mean (SEM) of 4 donors. **(B)** Comparison of experimental and calculated GAG per DNA contents of cells pellets. Calculated GAG per DNA content was calculated as per equation given in Methods and Materials sub-section Biochemical analysis. Data are mean ± standard error of mean (SEM) of 4 donor pairs. One-way analysis of variance (ANOVA) with Tukey’s multiple comparison post-tests: (*) indicates p <0.05, (**) indicates p < 0.001 and (***) indicates p < 0.0001 for statistical significance of comparison.

Firstly, we established that chondrogenic co-culture of MCs and BMSCs resulted in a synergistically enhanced cartilaginous matrix formation relative to mono-cultures of MCs and BMSCs as previously reported [21]. After a total of 17 days of cell culture in 3D cell pellet format in the presence of TGFβ3 supplemented chondrogenic media, primary human MCs, passaged 2 expanded BMSCs or in co-culture with each other at 1 to 3 ratio, respectively, formed hyaline-like cartilaginous tissue with varied staining intensity for GAG (as detected by Safranin O stain) and collagen II (Figure 1A). Qualitatively, the GAG and collagen II staining intensity was least in mono-cultured meniscus cell pellets and highest in co-cultured cell pellets of MCs and BMSCs. Biochemical analysis for the accumulated GAG amount after normalization β to the cellular DNA content, revealed statistically significant differences between the GAG content of co-cultured and mono-cultured pellets of MCs (p value of 0.005; Figure 2A). The GAG/DNA content was 3.40 ± 0.19 μg/μg (for MCs pellets), 6.43 ± 0.32 μg/μg (for co-cultured MCs:BMSC pellets) and 6.42 ± 0.30 μg/μg (for mono-cultured BMSC pellets) (Figure 2A; Table 2).

**Table 2:** GAG/DNA contents of pellets before and after treatment with IL-1β

**Pellets**	GAG/DNA (μg/μg) ± SEM*	**IL-1β (500 pg/ml)**
MC	3.40 ± 0.19	-
MC:BMSC	6.43 ± 0.32	-
BMSC	6.42 ± 0.30	-
MC+	2.87 ± 0.46	+
MC:BMSC+	6.11 ± 0.31	+
BMSC+	6.30 ± 0.56	+

*Standard error of mean (SEM).

### Effect of IL-1β on cartilaginous tissue formation in co-cultures of MCs and BMSCs

Addition of IL-1β (500 pg/ml) on day 14 of the 17 days of chondrogenic pellet culture resulted in tissues with reduced Safranin O staining appearance relative to control pellet cultures without IL-1β treatment (Figure 1A and B). In contrast, the collagen II staining intensity appeared similar between control and experimental pairs although there was some evidence of altered distribution of the matrix protein within pellets. Biochemical analysis for GAG/DNA content confirmed the reduced GAG staining intensity in pellets formed from mono-cultured MCs relative to mono-cultured pellets of BMSCs or co-cultured pellets of MCs and BMSCs (Table 2). Although the GAG/DNA value for pellets from mono-cultured MCs (2.87 ± 0.46 μg/μg) and co-cultured cells (6.11 ± 0.31 μg/μg) were lower relative to control pellets without IL-1β treatment, the values were not significantly different (p values of 1.00 and 0.98, respectively) from that of control pellets (Figure 2A). There was no significant difference between the GAG/DNA contents of BMSCs pellets treated with or without IL-1β (p value 1.00; Figure 2A).

Based on the experimental GAG/DNA values of mono-cultured MCs and BMSCs, the calculated GAG/DNA content of co-cultured pellets of MCs and BMSCs was determined as a linear function of the proportion of MCs and BMSCs in the co-cultured pellets. In the absence of IL-1β, the calculated GAG/DNA content was 5.66 ± 0.05 μg/ug, while in the presence of IL-1β the calculated GAG/DNA content was 5.44 ± 0.04 μg/ug. The experimentally determined GAG/DNA contents of the co-cultured pellets were 6.43 ± 0.32 μg/ug, in the absence of IL-1β, and 6.11 ± 0.31 μg/ug after treatment with IL-1β. The experimental GAG/DNA value for co-cultured pellets in the absence of IL-1β was significantly higher than calculated (1.14-fold; p value of 0.001). Similarly, there was a significant difference (1.12-fold; p value of 0.002) between the calculated and experimental GAG/DNA values of the co-cultured pellets after IL-1β treatment i.e. 5.44 ± 0.04 μg/ug (calculated) vs. 6.11 ± 0.31 μg/ug (experimental) (Figure 2B).

### Effect of IL-1β on gene expression profile of cultures of meniscus cells and BMSCs

**Figure 3:**
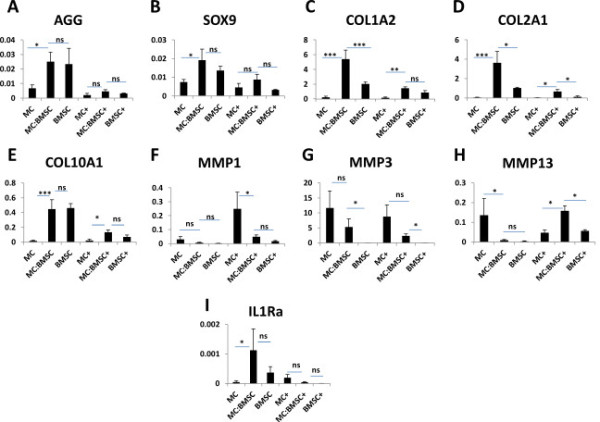
**Gene expression analysis via quantitative real time RT-PCR.** mRNA gene expression analysis via SYBR Green detection was used to evaluate gene expression of: **(A)** aggrecan, **(B)** sox9, **(C)** collagen I (*COL1A2*), **(D)** collagen II (*COL2A1*), **(E)** collagen X (*COL10A1*) **(F)** matrix metalloproteinase-1 (MMP-1), **(G)** matrix metalloproteinase −3 (MMP3), **(H)** matrix metalloproteinase-13 (MMP13), and **(I)** interleukin 1 receptor antagonist (IL1Ra) in pellets from MCs, MC:BMSCs and BMSCs after 17 days of culture in serum free chondrogenic medium containing TGF-β3, dexamethasone and ascorbate. In the last 3 days of pellet culture, pellets were either cultured in the presence (signified by +) or absence of IL-1β. Each data point represents the mean ± SEM. One-way analysis of variance (ANOVA) with Tukey’s multiple comparison post-tests was performed using SPSS version 20 and statistical significance level indicators are: *p <0.05, **p <0.01; *** *p* <0.0001. No significant (ns) difference. All marker gene expression data was normalized to expression of human β-actin; *y*-axis.

We evaluated the expression of a panel of genes (i.e. aggrecan, collagens I and II, Sox9 that we had previously reported to be synergistically up-regulated in chondrogenic co-cultures of meniscus cells and bone marrow mesenchymal stromal cells, and the expression collagen X that had been shown to be down-regulated in co-cultures of meniscus cells and BMSCs [21,33,34]. Additionally, we investigated the expression of a panel of catabolic genes, MMP-1, MMP-3 and MMP-13, which have been reported to be modulated at both the mRNA and protein levels by IL-1β in chondrocytes to [42]. Finally, we investigated the expression of IL1Ra as it had been shown to mediate the anti-inflammatory and anti-fibrotic response of BMSCs to IL-1α in the lungs of mice, and inhibition of IL-1β mediated matrix degradation in human intervertebral discs [43,44]. The following observations were made in pellets cultured in the absence or after short term treatment of pellets with IL-1β: (1) the transcript expression of aggrecan and Sox9 were significantly upregulated in co-cultured pellets than in mono-cultured pellets of MC in the absence of IL-1β. The expression of aggrecan and Sox9 in the co-cultured pellets was not significantly different from their expression in pure BMSC pellets. However, after short-term treatment of all pellets with IL-1β, the level of expression of both genes declined (relative to pellets without IL-1β treatment) to levels that were not statistically different between the different pellet groups (Figure 3A and B); (2) in the absence of IL-1β, the expression of collagen I was significantly higher in co-cultured pellets relative to its expression in mono-cultured pellets. After IL-1β treatment, the expression of collagen I declined in all pellets groups, however, its expression (although decreased) remained significantly higher in the co-cultured pellets than in pure MC pellets. In contrast, collagen I’s expression was similar in co-cultured pellets and pure BMSCs pellets after IL-1β treatment (Figure 3C). Similarly, the expression of collagen II was significantly higher in the co-cultured pellets compared to its expression in either pure MC or pure BMSC pellets in the absence of IL-1β. After treatment of all pellets with IL-1β, the expression of collagen II declined relative to control pellets without IL-1β treatment but its expression remained significantly higher in the co-cultured pellets relative to its expression in pure MC and pure BMSCs pellets (Figure 3D); (3) in the absence of IL-1β, the level of collagen X expression in pure BMSC and co-cultured pellets was significantly higher (26-fold; p value of 0.001) than its expression in pure MC pellets. In contrast, the expression of collagen X decreased to the same level in both co-cultured and mono-cultured pellets of BMSC after IL-1β treatment. Collagen X expression remained at the same level of expression in MC pellets before and after treatment with IL-1β (Figure 3E); (4) in the absence of IL-1β, the level of MMP-1 expression was similar in all pellets. However, after IL-1β treatment, we observed a statistically significant (p value of 0.02) increase in MMP-1 expression in mono-cultured MC pellets relative to other pellets (Figure 3F). Additionally, MMP-1 expression was significantly up-regulated (21-fold; p value of 0.006) in co-cultured cell pellets after IL-1β treatment relative to co-cultured controls without IL-1β treatment (Figure 3F). In contrast, the level of MMP-3 expression remained the same in all pellets before and after treatment with IL-1β (Figure 3G). The expression of MMP-13 in pure MC pellets was not significantly modulated by IL-1β. However, IL-1β increased the expression of MMP-13 in pure BMSC and co-cultured pellets (Figure 3H). The expression of IL1Ra was highest in co-cultured pellets without IL-1β treatment (Figure 3I). After treatment with IL-1β, the expression of IL1Ra declined significantly by 24-fold in co-cultured pellets relative to its expression in co-cultured pellets without IL-1β treatment. IL-1β did not significantly modulate the expression of IL1Ra in pure MC or pure BMSC (Figure 3I).

## Discussion

Previously we showed that co-culture of primary human MCs with BMSCs formed tissue with a fibrocartilage phenotype and a synergistically enhanced GAG matrix content relative to mono-cultured MCs and mono-cultured BMSCs after pellet culture in chondrogenic media [21]. Similar findings were also reported by Cui *et al.*[34]. In this study we evaluated and compared the response of co-cultured MCs and BMSCs in 3D pellets, as well as mono-cultured MC and mono-cultured BMSC pellets, to short-term treatment of IL-1β. In the absence of IL-1β, we observed significant increase in the GAG matrix-forming capacity of co-cultured MCs and BMSCs pellets relative to control mono-cultured pellets of MCs and BMSCs. In the presence of IL-1β, the GAG matrix-forming capacity of both co-cultured cells and mono-cultured MCs decreased slightly but not significantly compared to their control without IL-1β. Similarly, the GAG matrix-forming capacity of mono-cultured BMSCs decreased slightly (relative to control BMSCs) after treatment with IL-1β. Based on the proportion of MCs and BMSCs in the co-cultured pellets and the GAG matrix per DNA of mono-cultured MCs and mono-cultured BMSCs, the experimentally determined amount of GAG matrix per DNA in the co-cultured pellets significantly exceeded expectation in the absence of IL-1β. A similar outcome occurred after short term treatment of co-cultured pellets with IL-1β; the experimentally determined amount of GAG matrix per DNA in the co-cultured pellets exceeded the expected GAG per DNA content. This finding suggested that the synergistic interplay between MCs and BMSCs was retained after short term treatment of pellets with IL-1β. While the mechanism underlying the interplay of MCs and BMSCs in the presence of IL-1β was not investigated in this study, it appears to be associated with increased transcription of MMP-1 and MMP-13, and perhaps MMP-1 and MMP-13 mediated matrix remodelling. Having said that, based on histological and gene expression findings in this study, it is reasonable to speculate that the degree of response of the co-cultured pellets to IL-1β treatment is dependent on the differential effects of IL-1β on the MCs and BMSCs components of the co-cultured pellet. For example, safranin O staining was lost in mono-cultured MCs pellets after IL-1β treatment, while mono-cultured BMSCs pellets retained some of its positive safranin O staining for GAG matrix. Additionally, after IL-1β treatment, there was a highly significant increase (7-fold) in MMP-1 expression in mono-cultured MCs relative to controls, while IL-1β had little or no inductive effect on mono-cultured BMSCs’ MMP-1 expression. This suggests that the significant up-regulation in MMP-1 expression in co-cultured pellets was due to the MCs component of the co-culture. Furthermore, IL-1β modulated the expression of MMP-13 in the BMSCs component (but not in MC component) of the co-cultured pellets and perhaps more synergistically in the presence of MC as evaluated in the co-cultured pellets. Nevertheless, our findings are consistent with the effect of IL-1β on engineered tissues from meniscus-like cells (i.e. cell-culture expanded nasal and articular chondrocytes) that MMP-1 expression was enhanced and accompanied by decreased GAG matrix-forming capacity [42,45]. However, it was surprising that IL-1β did not modulate the expression of MMP-3 in mono-cultured pellets of MCs or in the co-cultured pellets since Lemke *et al.*[46], although with a different IL-1 isoform, had reported increased MMP-3 mRNA expression post IL-1α treatment of bovine meniscus explants.

Additionally, our data showed that the GAG/DNA contents of mono-cultured pellets of MCs declined after treatment with IL-1β and this finding was consistent with a reduction or loss of safranin O staining. However, a similar finding was absent in mono-cultured BMSCs and in co-cultured MC and BMSC pellets where a relatively more visible and positive staining of safranin O remained post IL-1β treatment. The reason for this is unclear but may be associated with a significantly higher expression of interleukin 1 receptor antagonist (IL1Ra) in the co-cultured or BMSC pellets relative to MC pellets prior to IL-1β treatment since IL1Ra has been implicated in mitigating the pro-inflammatory effects (i.e. inflammation and fibrosis) of IL-1α in mice lungs after treatment with BMSCs [44]. While the potential involvement of IL1Ra needs further investigation, our finding that collagen I expression was down-regulated in BMSCs after treatment with IL-1β provides supporting evidence of its involvement since reduction of collagen I expression is a characteristic feature of anti-fibrosis [47].

Although the fibrogenic (*COL1A2*) and chondrogenic (*AGG*, *COL2A1* and *Sox9*) mRNA expression of the co-cultured cell pellets declined after IL-1β treatment, our data demonstrated that a synergistically enhanced chondrogenic GAG matrix formation remained in the co-cultured pellets even after treatment with IL-1β. The reason for this disparity may be a time lag between transcription and translation of genes [48], or alternatively that the 72 hour treatment of pellets with IL-1β was not a sufficient timeframe to adequately evaluate the turnover of GAG matrix accumulated in the co-cultured pellets. Nonetheless, our data is consistent with previous findings that IL-1β reduces the abundance of mRNAs encoding COL2A1, aggrecan and Sox9 [49]. Moreover, our data aligns with previous findings that IL-1β suppresses collagen X expression in chondrocytes [50]. Although, for some unknown reason the expression of collagen X was not suppressed in the absence of IL-1β post co-culture of MC and BMSCs as we and others have previously reported [33,34]. On the whole, it is probable that the down-regulation of the chondrogenic genes in our study by IL-1β was mediated by activation of nuclear factor-KappaB (NF-kB), a transcription factor known to suppress the synthesis of Sox9 through post-transcriptional processes that destabilize Sox9 mRNA [51,52].

## Conclusions

This study showed that: (1) chondrogenic co-cultures of primary MCs and BMSCs led to the formation of a neo-tissue that was characterized by enhanced production of GAG matrix relative to mono-cultures of pure MCs and pure BMSCs; (2) short term treatment of the chondrogenic co-cultures of MCs and BMSCs with IL-1β resulted in a neo-tissue characterized by a GAG matrix content that was consistent with more than the sum of the GAG matrix produced by the proportion of MCs and BMSCs in the co-cultured pellets; (3) the tissue formed by co-cultured MCs and BMSCs even after treatment with IL-1β had a phenotype (based on collagen I and II gene expression data) that was more consistent with meniscus fibrocartilage than tissues formed from mono-cultures of MCs and BMSCs. Thus, our findings strengthen the use of co-cultured primary MCs and BMSCs as a combinatorial cell source for meniscus tissue engineering.

## Abbreviations

BMSCs: Bone marrow mesenchymal stromal cells; cDNA: Complementary deoxyribonucleic acid; COL1A2: Type I collagen α2 chain; COL2A1: Type II collagen α1 chain; COL10A1: Type X collagen α1 chain; ECM: Extracellular matrix; FGF-2: Basic fibroblast growth factor; GAG: Glycosaminoglycan; IL-1β: Interleukin 1 beta; IL1Ra: Interleukin 1 receptor antagonist; MCs: Meniscus cells; MMP-1: Matrix metalloproteinase 1; MMP-3: Matrix metalloproteinase 3; MMP-13: Matrix metalloproteinase 13; mRNA: Messenger ribonucleic acid; MSCs: Mesenchymal stromal cells; NF-kB: Nuclear factor-KappaB; OA: Osteoarthritis; qRT-PCR: Quantitative real-time polymerase chain reaction; RNA: Ribonucleic acid; SOX9: Sry-related HMG box-9; TGF-β3: Transforming growth factor -β3.

## Competing interests

The authors declare that they have no competing interests.

## Authors’ contributions

AC, LWB, AMS: performed experiments and data acquisition. NMJ: responsible for tissue procurement, data analysis and manuscript writing. ABA: conceived and designed the study, data acquisition and analysis, manuscript writing and supervision of entire study. All authors read and approved the final manuscript.

## Pre-publication history

The pre-publication history for this paper can be accessed here:

http://www.biomedcentral.com/1471-2474/14/216/prepub
